# MicroRNAs in colorectal carcinoma - from pathogenesis to therapy

**DOI:** 10.1186/s13046-016-0320-4

**Published:** 2016-03-10

**Authors:** Yudan Chi, Dongming Zhou

**Affiliations:** Vaccine Research Center, Key Laboratory of Molecular Virology & Immunology, Institut Pasteur of Shanghai, Chinese Academy of Sciences, Shanghai, 200031 China

**Keywords:** MicroRNA, Colorectal carcinoma, Pathogenesis, Diagnosis, Cancer therapy

## Abstract

**Background:**

Acting as inflammatory mediators, tumor oncogenes or suppressors, microRNAs are involved in cell survival, death, epithelial–mesenchymal transition and metastasis, etc. Investigating the communication between microRNAs and tumorigenesis is critical to our understanding of the pathogenesis of multiple disease states.

**Main body:**

Currently, colorectal carcinoma (CRC), one of the most common malignancies worldwide, has a poor prognosis due to lack of an effective therapeutic option. Increasing evidence has identified altered profiles and regulatory potential of microRNAs in conditions related to environmentally-caused colorectal inflammation and colitis-associated cancer. Many studies have shed light on a more thorough understanding of the function and distribution of microRNAs in CRC initiation and emergence. However, the molecular mechanisms by which microRNAs modulate cellular processes still need to be further elucidated and may offer a foundation for evaluating microRNA-based therapeutic potential for CRC in both animal models and clinical trials.

**Conclusion:**

In this review, the roles and mechanisms of microRNAs involved in CRC from pathogenesis to therapy are summarized and discussed, which may provide more useful hints for CRC prevention and therapy.

## Introduction

MicroRNAs (miRNAs) are endogenously expressed non-coding RNAs, 18–25 nucleotides in length, which silence target mRNAs by mediating translational repression [1–4]. The miRNA biogenesis pathway includes multiple steps. Initially, pri-miRNAs (primary-miRNAs) containing a hairpin structure are transcribed by the RNA polymerase II which is responsible for mRNA expression. These pri-miRNAs are then cleaved into 60–70 base pairs long precursor miRNAs (pre-miRNAs) by the RNase III Drosha. Then, the pre-miRNAs are transported from the nucleus to the cytoplasm by Exportin-5/RanGTP and further processed by Dicer to form a short double-stranded miRNA duplex. Generally, only one strand of this miRNA duplex is degraded, while the other strand is released as a mature miRNA. Subsequently, this miRNA is integrated into RISC (RNA-induced Silencing Complex) to trigger degradation and translational repression of the target mRNA [5, 6].

MiRNAs play a key role in many crucial biological processes such as cell proliferation, cell differentiation and apoptosis [7–10]. In the past 20 years, more evidence has emerged showing that miRNAs are also involved in cancer development. Aberrant expression of miRNAs is detected in various types of cancer including breast cancer, lung cancer, pancreatic cancer, colorectal carcinoma and ovarian cancer [11–14]. MiRNAs regulate expression of many known oncogenes and tumor suppressor genes in cancer pathogenesis [15, 16]. Studying the specific function of miRNAs in human carcinogenesis will help to identify new targets for cancer research, diagnosis and treatment.

CRC is the second most common malignancy in women, and the third in men, worldwide. More than 1 million new cancer-related cases and 600,000 deaths are expected to occur each year [17, 18]. Many risk factors associated with CRC include excessive alcohol use, obesity, older age, some genetic mutations and chronic intestinal inflammation. Generally, CRC consists of inflammatory colitis-associated cancer (CAC) and non-inflammatory adenomatous CRC. Inflammatory bowel disease (IBD) is always associated with CAC and about 20 % IBD patients develop CAC 30 years after the onset of disease [19]. Like other types of cancer, colorectal carcinogenesis is a multistep and complex process including tumor initiation, promotion and metastasis. Recent studies have revealed that the pathogenic mechanisms of CRC depend on several signaling pathways, including the p53, PI3K, RAS, MAPK, EMT transcription factors, and Wnt/β-catenin pathways. Furthermore, it has become increasingly clear that miRNAs regulate these pathways involved in the CRC pathogenesis (Table 1). For example, reduced expression of miR-143 contributes to CRC development through derepressing KRAS expression [20]. MiR-133a regulates CRC by inhibiting MAPK pathways [21].

**Table 1:** miRNA involvement in CRC development, diagnosis and therapy

miRNAs	Function	References
miR-324-5p,miR-21,miR-181b-1,miR-146,	Involved in regulation of NF-KB signaling in inflammation-related CRC	[30–50]
miR-126,miR-122,miR-192,miR-495,miR-671,		
miR-106b,miR-30c,miR-130a		
miR-16,miR-218,miR-34a	Involved in the cell proliferation and survival in CRC development	[51–59]
miR-34a,miR-148,miR-339-5p,miR-504,miR-23a,miR-129,miR-365,miR-345	Involved in the cell death in CRC development	[62–74]
miR-29a,miR-29b,miR-29c,miR-200a,miR-200b,miR-200c,miR-141,miR-429,miR-132,miR-192,	Involved in the EMT in CRC development	[79–95]
miR-335,miR-34a		
miR-214,miR-155,miR-483,miR-133a,miR-145,	Involved in the tumor invasion and metastasis in CRC development	[96–112]
miR-21,miR-92a,miR-17-5p,miR-221,miR-499-5p,miR-182		
miR-92a-3p,miR-29a,miR-17-3p,miR-221,	Involved in the clinical diagnosis of CRC	[123–129]
miR-19a-3p,miR-223-3p,miR-422a,miR-143,		
miR-145,miR-21,miR-106a,miR-92a, miR-144		
miR-135b,miR-27b,miR-4689,miR-483-5p,	Involved in the therapy of CRC	[131–152]
miR-551a,miR-34a,miR-22,miR129,miR-365,		
miR-143,miR-21,miR-23a,miR-124		

To summarize the roles and mechanisms of miRNAs involved in CRC, it is important to be pointed out that CRC is a heterogeneous cancer including both colon cancer and rectal cancer while numerous literatures misused the term CRC in many cases [22]. From the clinical point of view, colon cancer and rectal cancer should be treated separately. Unfortunately, the majority of previous studies failed to separate these two entities. Here we distinguish the respective miRNAs in these two cancers based on the related references although very limited data about miRNAs in the rectal cancer are available [23–29].

### Role of miRNAs in CRC

#### Inflammation

**Fig. 1:**
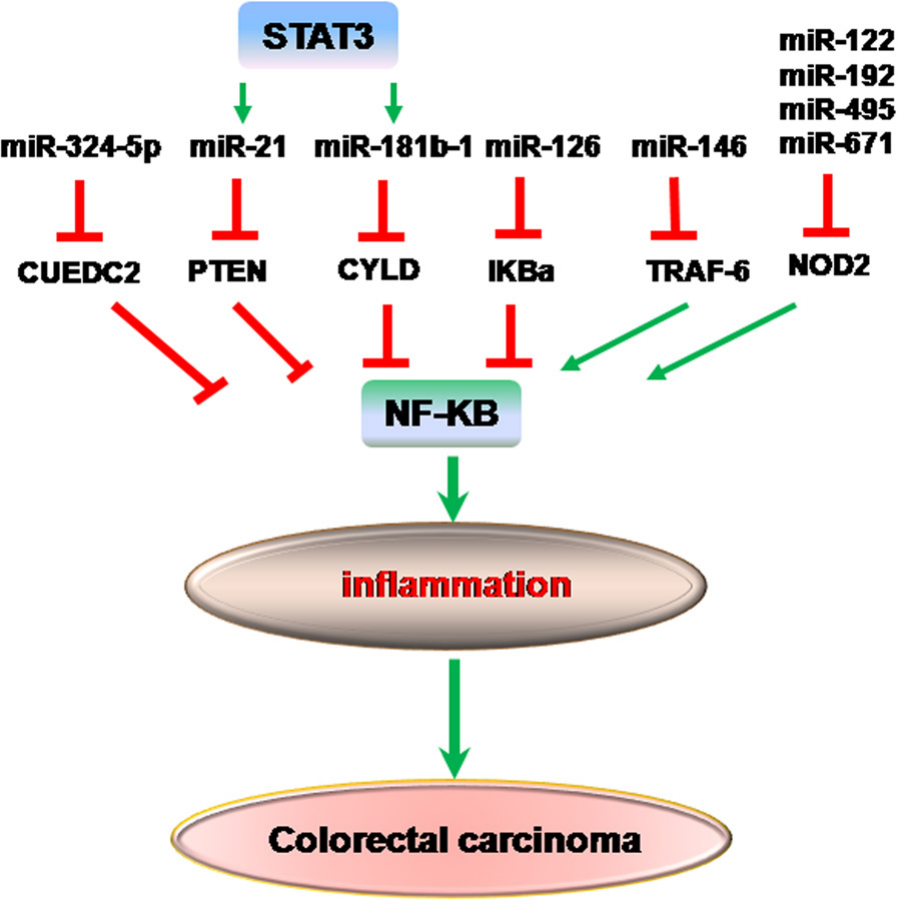
MiRNAs in inflammation-related colorectal carcinoma. NF-KB signaling maintained constitutive activation of pro-inflammatory pathways as essential components during carcinogenesis. Many miRNAs target NF-κB signaling molecules to inhibit (miR-324-5p, miR-21, miR-181b-1, miR-126) or promote (miR-146, miR-122, miR-192, miR-495, miR-671) inflammatory response in the development of colorectal carcinoma

Various environmental causes contribute to colorectal inflammation, including microbial infections, metabolic disorders, toxins and dietary factors [30–32]. Growing evidence indicates that a plethora of miRNAs will target inflammatory signaling molecules to induce or inhibit chronic inflammation and inflammation-related cancer (Fig. 1) [19, 33].

The nuclear transcription factor, NF-κB and signal transducer and activator of transcription 3 (STAT3) maintain constitutive activation of pro-inflammatory pathways as essential components in the development of CAC tumors [34–36]. Targeting negative regulators of NF-κB signaling through miRNAs, e.g., miR-324-5p-CUEDC2, miR-21-PTEN, miR-181b-1-CYLD, miR-146-TRAF and miR-126-IKBa, result in inflammation hyperresponsiveness and tumorigenesis. MiR-324-5p, a new CRC-associated miRNA, regulates CUEDC2 levels during monocyte to macrophage differentiation [37]. Elevation of miR-324-5p levels results in decreased expression of CUEDC2 in macrophages infiltrated in mouse colon tumors and isolated from fresh colon tumor samples, which produces excess tumor-promoting cytokines and promotes pathogenic progress of CRC. The function of STAT3 in cellular transformation involves the direct activation of miR-21 and miR-181b-1 transcription by binding multiple sites in the miRNA promoter [38]. Overexpression of miR-21 or miR-181b-1 is sufficient to induce a stable transformed state by directly targeting PTEN and CYLD expression, respectively, which in turn activates NF-κB pathway. MiRNAs targeting NOD2, such as miR-122, miR-192, miR-495, and miR-671 decrease the pro-inflammatory cytokines by regulating the activation of NF-κB pathway [39, 40]. However, miR-122 is significantly increased with the stimulation of TNF-α and induces an increase in the intestinal epithelial tight junction permeability in vitro and in vivo [41]. Thus, the controversial role of miR-122 in the development of IBD should be further explored.

In accordance with previous works, miR-21 levels were often higher in inflammation and CRC than that of normal tissue [42–44]. MiR-21 is upregulated in IBD and acute intestinal obstruction (AIO) patients. In miR-21 knockout mice suffering from dextran sulfate sodium salt (DSS)-induced fatal colitis, the survival rate is improved and the ameliorative inflammatory response better protects against inflammation and tissue injury [45]. Also, miR-21 deletion exacerbates CD4+ T-cell-mediated models of acute and chronic DSS and TNBS colitis. In addition, some evidence indicates that miR-21 plays a pro-inflammatory role in IBD by impairing intestinal barrier function [46]. The increase in intestinal permeability and epithelial cells apoptosis induced by DSS are attenuated in miR-21 knockout mice.

Autophagy, involved in recycling cellular organelles for maintaining homeostasis, is considered to clear intracellular microorganisms [47]. The impairment of autophagy results in intestinal epithelial dysfunction and contributes to IBD pathogenesis [48]. Inflamed mucosae from subjects with active CD have higher miR-106b and lower ATG16L1 levels indicating an altered antibacterial activity that is mediated by miR-106b which subsequently affects the outcome of intestinal inflammation [49]. MiR-106b may target ATG16L1 and reduce the level of autophagy in HCT116 cells and inhibit autophagy–dependent clearance of CD-associated adherent-invasive Escherichia coli (AIEC) in epithelial cells. Another study showed the expression of miR-30c and miR-130a were inversely correlated with ATG5 and ATG16L1 in intestinal epithelial cells. The inhibition of the activity of autophagy by miRNAs promotes the persistence of AIEC and the production of pro-inflammatory cytokines [50].

#### Cell survival

Cellular proliferation and survival have crucial roles in the process of carcinogenesis. Abnormal expression of miRNAs regulates CRC development via targeting several cell cycle regulators, including survivin and cyclins. Survivin is a direct target of miR-16 [51]. MiR-16 represses CRC cell growth and induces cell apoptosis by regulating the p53/survivin signaling pathway. These observations suggest that survivin is mainly expressed during the G2/M phase of the cell cycle and therefore inhibiting survivin expression can lead to defective cytokinesis and cell cycle arrest at G2/M phase [52]. Among the other miRNAs that control cell cycle progression, miR-218 induces cell cycle arrest in the G2 phase of colon cancer cells by suppressing cyclin-dependent kinase4 (CDK4) and upregulating the level of p53 [53]. A recent study conducted by Cai et al. demonstrated that miR-144 inhibited cell proliferation in rectal cancer cell line SW137 and SW1463 by downregulating Rock-1. However, the aberrant expression of miR-144 is only present in the rectal cancer but not in the colon cancer [54].

The role of miR-34a to CRC development was already clarified with miR-34a inhibiting colon cancer cell proliferation by downregulating the E2F pathway and resulting in accumulation of p53 and p21 [55]. Recent studies have revealed that PAR_2_ promotes cancer cell proliferation through the activation of EGFR, MAPK and other survival signals and promotes the accumulation of Cyclin D1 which plays important roles in tumorigenesis [56, 57]. Further investigations show that miR-34a mediated PAR_2_-induced proliferation and inhibition of miR-34a partially restores the activation of Cyclin D1 induced by PAR_2_ deficiency. Colon cancer stem cells (CCSCs) retain the self-renewal capacity and less limiting proliferative potential while being substantially resistant to most conventional anticancer therapies [58]. Moreover, various conserved pathways, such as Notch and Wnt, as a complex crosstalk network between CCSCs and microenvironment, are regulated in CRC. MiR-34a in the regulation of CCSCs self renewal is involved in the suppression of Notch signaling, which contributes to asymmetric cell division of stem cells [59]. Altogether, this finding reveals a unique miR-34a-regulated mechanism of the toggle switch necessary for Notch bimodality that converts noisy signals into unambiguous states for robust cell-fate decisions in CCSCs.

#### Cell death

The p53 protein is a transcription factor that is activated in response to cellular stresses to inhibit cell proliferation and stimulate cell death [60]. Disruption of the p53 pathway can promote tumorigenesis [61]. MiR-34a mediated inhibition of SIRT1 expression leads to apoptosis due to the increase of acetylated p53 formed a positive feedback loop of miR-34a and p53 [62]. Moreover, transient introduction of miR-34a into SW480 cells contributes to a severe decrease in migration and invasion by upregulating acetylated p53 and p21 [63]. It also suggests that an overexpression of miR-34a induces cell growth arrest and senescence-like phenotypes through upregulating the p53 pathway [55]. The increase of p55PIK in CRC can accelerate cell cycle progression by interacting with retinoblastoma protein or proliferation cell nuclear antigen [64, 65]. The introduced miR-148b, by suppressing p55PIK abolishes cell proliferation and cell cycle progression in CRC [66]. Furthermore, p53 directly activates the transcription of miR-148 which negatively regulates p55PIK expression. A reduction of miR-339-5p expression has been reported in colorectal cancer and is associated with poor prognosis in cancer patients [67, 68]. MDM2, a key negative regulator of p53 is repressed by miR-339-5p [69]. After downregulation of MDM2 by miR-339-5p, the growth of colorectal xenograft tumors is inhibited in a p53-dependent manner. The function of miR-504, that is inhibiting p53 expression, reduces cell cycle arrest and promotes tumorigenicity in vivo [70].

Apoptosis is also controlled by various networks of miRNAs. Several researches have described that proapoptotic protein can be suppressed by the overexpression of miRNAs. For example, the human homolog of the *Caenorhabditis elegans* cell death protein CED-4, APAF-1, is controlled by miR-23a to repress the activity of caspases-3,-7 and-9 [71]. The increased miR-23a antisense can induce the apoptosis of HCT116 and HT29 cell lines, under the 5-FU treatment. It is also found that miR-23a is up-regulated in 5-FU-treated HC.21 and C22.20 cells [72]. Conversely, miR-129 can trigger apoptosis by suppressing Bcl2, an anti-apoptotic protein [73]. The Intrinsic apoptotic pathway is activated by cleavage of caspae-9 and-3. Besides, the transfection of miR-129 in RKO cells and HCT116 cells causes cell cycle arrest in G1 or G2 phase. In human CRC tissues, miR-129 is significantly decreased in patients with stage 3 and stage 4 tumors. The other miRNAs, such as miR-365 and miR-345, also affect the antitumor capability, respectively by targeting the anti-apoptosis protein of Bcl2 and Bcl2-associated athanogene 3 (BAG3) [74].

#### MiRNAs and EMT

**Fig. 2:**
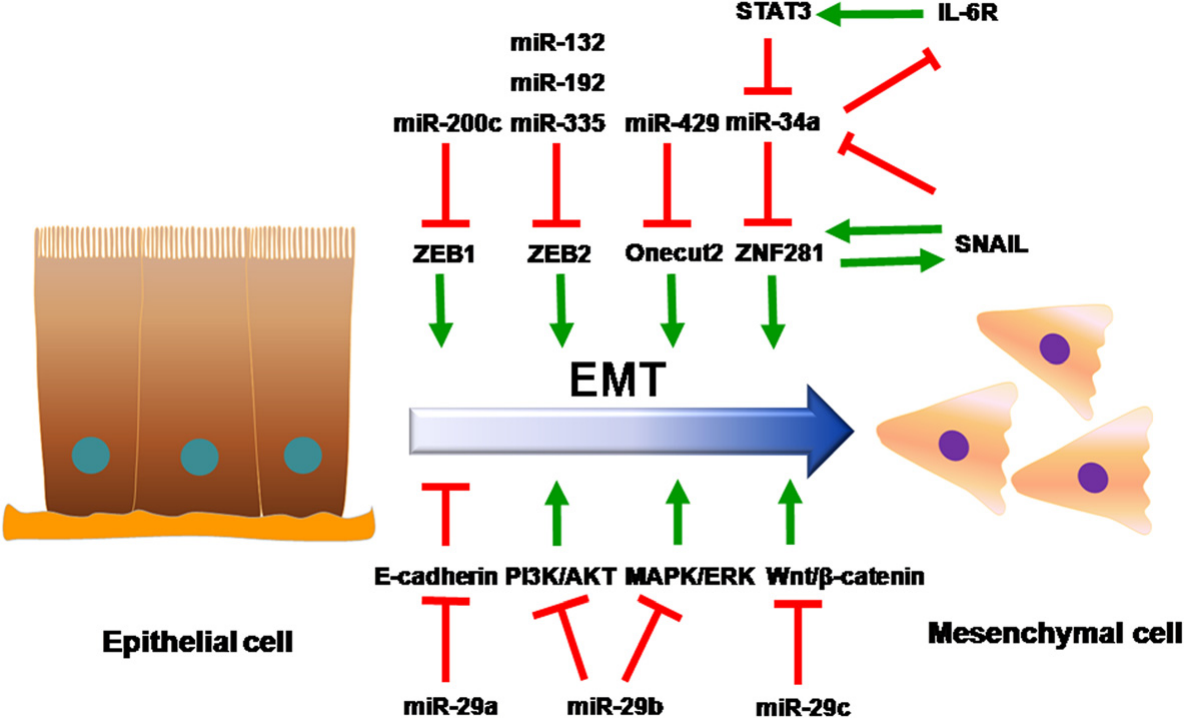
Regulation of epithelial mesenchymal transition (EMT) in colorectal carcinogenesis by miRNAs. Many miRNAs, such as miR-29b, miR-29c, miR-200c, miR-34a, regulate EMT by suppressing EMT-related transcription factors and signaling pathways. The other miRNAs, such as miR-29a, promote EMT in colorectal carcinogenesis

Epithelial-to-mesenchymal transition (EMT) is involved in multiple biological processes including gastrulation, neural tube formation, tissue regeneration, and organ fibrosis [75]. EMT is an important factor in tumor metastasis undergoing a number of biochemical changes, including the decrease in epithelial cell-surface markers and cytoskeleton components, and the increase in mesenchymal markers and specific transcription factors [76–78]. Given miRNAs-regulated EMT via targeting E-cadherin and other molecules, it is likely that miRNAs play a crucial role in colorectal carcinogenesis (Fig. 2). The highly conserved pathway of Wnt/β–catenin signaling is constitutively activated in CRC. Wnt signaling is regulated by abnormal β–catenin activation associated with E-cadherin expression [79]. Also, miR-101, miR-224 and miR-574-5p can affect CRC malignant features by regulating Wnt/β–catenin signaling [80–82].

Recent studies have demonstrated that the members of the miR-29 family (miR-29a, miR-29b and miR-29c) are involved in the tumor progression by regulating EMT. MiR-29c is dramatically downregulated in CRC tissues and suppresses EMT in vitro, hence it has a role in cell migration and invasion by negatively regulating the Wnt/β-catenin signaling pathway [83]. Similarly, miR-29b suppresses EMT and plays an important role in cell migration and invasion by negatively regulating the MAPK/ERK and PI3K/AKT pathways [84]. However, overexpression of miR-29a promotes cell invasion by inhibiting E-cadherin expression [85].

The importance of the miR-200 family (miR-200a, miR-200b, miR-200c, miR-141 and miR-429) for EMT is not limited to colon carcinogenesis, because it has been widely demonstrated in various tumors [86, 87]. Indeed, restoration of miR-200c inhibits migration and invasion in various CRC cell lines via directly targeting ZEB1, the transcriptional repressor of E-cadherin [88, 89]. There is another evidence that miR-429 reverses TGF-β-induced EMT by interfering with Onecut2 in SW620 and SW480 cells [90]. In addition, ZEB2 as a direct target of miR-132, miR-192 and miR-335 has been shown to regulate metastasis [91–93]. A significant decrease of these three miRNAs is associated with distant metastasis and advanced stage tumors. A few researches have indicated that miR-34a inhibits metastasis formation in CRC via EMT-regulating network in SNAIL/ZNF81 and IL6R/STAT3 [94, 95]. These studies implicate the components of miRNA-regulating networks in EMT with traits associated with metastasis formation in CRC.

#### Invasion and metastasis

Over 70 % of CRC patients harboring liver metastasis die due to the lack of effective therapeutics. Some miRNAs have been identified to suppress liver metastatic colonization in CRC patients. Nude mice in which ectopic miR-214 is expressed in CRC cells has a reduced amount of liver metastases, supporting the importance of intracellular dynamic regulation [96]. However, another crucial factor for metastasis is based on extracellular metabolic energetic [97]. Creatine kinase Brain (CKB) in liver metastatic cancer cells is released to the extracellular microenvironment converting ATP and creatine into phosphocreatine, which is imported into cancer cells to counteract hypoxic response. Both miR-155 and miR-483 targeting CKB, as endogenous suppressors of colon cancer metastasis decrease significantly the ATP levels, further to impair intracellular energetic requirements to establish a barrier to metastatic progression.

MiR-133a targets LASP1 and suppresses tumor growth and metastasis by inhibiting phosphorylation of the ERK/MEK signaling pathway [21]. Downregulation of miR-145 is detected in primary CRC tumors compared to normal tissues. MiR-145 inhibits proliferation, migration and invasion of SW620 and LoVo metastatic cell lines by targeting fascin-1 and results in a decrease in lung metastases in nude mice [98]. Conversely, another study reveals that increased miR-145 could improve migration and invasion of HCT-8 cells and is associated with lymph node metastasis of CRC while having no effect on proliferation [99]. In addition, miR-106b promotes colorectal cancer cell migration and invasion by directly targeting DLC1 [100].

PTEN, a tumor suppressor gene, is often lost in human cancers and a common target of miR-21, miR-92a and miR-17-5p in CRC [101–104]. The levels of miR-21 and miR-92a significantly correlate with lymph node metastasis and advanced TNM stage promoting cell migration via mediating the PTEN-dependent PI3K/AKT signaling pathway. Overexpression of miR-17-5p is responsible for chemo-resistance in a cohort study of 295 patients. In addition, miR-221, miR-145, miR-499-5p and miR-182 as novel candidate prometastatic miRNAs are significantly increased in lymph node-positive CRC patients by regulating suppressor genes (cyclin dependent kinase inhibitor, RECK, FOXO4 and PDCD4) [105–109].

The small GTPases Cdc42 is essential for intestinal stem cell division, survival and differentiation to maintain the homeostasis [110]. In addition, Cdc42 is required higher expression in human CRCs relating to poorly differentiated CRCs [111]. In the APC-mutant or β-catenin-mutant mice, Cdc42 reduction attenuates the tumorigenesis of mutant intestinal cells [112]. In the same way, human colorectal cancer with higher levels of Cdc42 activity was especially sensitive to Cdc42 blockade. Expression of miR-224 in CRC tissue specimens is significantly lower than in nontumor tissues and paired adjacent samples [113]. Ectopic expression of miR-224 inhibits the migratory ability of HCT116 cells, but the cell growth rate is less affected. Increased miR-224 suppresses CRC cell migration by diminishing Cdc42 and SMAD4 expressions and inhibiting the formation of actin filaments.

#### MiRNAs as clinical diagnosis

Given the invasive nature and expensive cost of current screening methods of CRC diagnosis including FOBT, CEA and colonoscopy, it is difficult to detect CRC early and efficiently [114–116]. As well as the importance of miRNAs in the CRC development, miRNAs could serve as potential biomarkers in CRC diagnosis based on the high degree of stability, specificity and sensitivity of miRNAs in the blood and stool [117–122].

Since the first study in 2008 reported cancer-specific miRNAs secreted in the blood among the different cancer types, a spectrum of miRNAs associated with CRC in the blood have been identified including miR-92a, miR-29a, miR-17-3p, miR-221, miR-19a-3p, miR-223-3p and miR-422a et al. [123–126]. Although ribonuclease exists in serum, circulating extracellular miRNAs are found in the blood of healthy and cancer-related patients. Chen et al. systematically identified specific patterns of serum miRNAs expression in several diseases including lung cancer, CRC, and diabetes [123]. This study showed that 69 miRNAs were detected in the blood of CRC patients but not in the normal serum. Unsuprisingly, many circulating miRNAs such as miR-221 are also present in the blood of lung cancer. Ng et al. discovered that both miR-92a and miR-17-3p were significantly upregulated in the plasma of CRC patients compared to normal plasma [125]. Zheng et al. recently identified four miRNAs panels (miR-19a-3p, miR-223-3p, miR-92a-3p and miR-422a) with a high diagnostic accuracy of CRC [124].

MiRNAs disturbance in the stool of CRC patients can also offer a possibility for a stool-base miRNA test as a common used method for CRC diagnosis. A number of studies support this potential diagnostic method of CRC by finding that many miRNAs are downregulated (miR-143, miR-145) or overexpressed (miR-21, miR-106a, miR-92a, miR-144) in the fecal samples of CRC patients compared to healthy subjects [127–130]. It is of interest to note that Wang et al. found that the expression level of miR-92a in the stool of CRC patients was significantly higher than control, which was very similar to the dysregulated expression of miR-92a in the blood [129]. Thus, miRNAs can be represented highly effective and accurate biomarkers for the future CRC diagnosis.

#### MiRNA-based therapies

Potential application of targeting miRNAs is increasing in gene therapy testing and preclinical studies. The development of mouse models generates key biological and molecular events based on human conditions. The efficacy of miRNA-mediated CRC therapy is following current technologies through various strategies.

Traditional preclinical mouse models of CRC induced by colitis - associated cancer (CAC) have been established with two drugs of azoxymethane (AOM) and DSS as the results of mutations containing PI3K, K-ras and catenin pathways. Another CDX2P-NLS Cre;Apc+/loxP (CPC; Apc) mouse model harbors a truncating mutation affecting one APC allele [131]. Compared to normal tissues, 57 miRNAs are aberrantly expressed in tumors in the AOM/DSS model while 35 miRNAs are aberrantly expressed in polyps from CPC; Apc mice [132]. Among the overexpressed miRNAs, miR-135b is consistently the highest expressed one in both models. High miR-135b expression is correlated with tumor stage and poor overall survival by analyzing 454 sporadic and 31 IBD-associated CRCs. The use of locked nucleic acid (LNA) anti-miR-135b induces apoptosis of SW480 cells while oligonucleotides specific silencing miR-135b effectively inhibited tumor proliferation in both mouse models. A study by Wu et al. indicated miR-135b mimics-transfected HCT-116 cells exhibited significantly increased migratory ability, while inverse effects were detected with the treatment of inhibitors [133]. Thus, miR-135b may be a promising therapeutic target in CRC treatment with improving specificity and limiting toxicity [134]. In addition to miR-135b, other oncogeic miRNAs are also potential candidates for CRC therapy. For example, successful knockdown of miR-21 by using LNA in SW480 cells and antisense oligonucleotide-based inhibition of miR-20a, miR-21,miR-31, miR-95, miR-675 in SW480, SW620, and HCT116 cells showed potential value for future translational treatment [135–139]. Although LNA and antisense oligonucleotide are efficient in blocking oncogenic miRNA, some novel approaches like miRNA sponge, miRNA masking and small molecule inhibitors are emerging. Jung and colleagues recently reported the use of miRNA sponges in human breast cancer cell lines [140]. They demonstrated a multi-potent miRNA sponge that simultaneously inhibits four oncogenic miRNAs including miR-21, miR-155,miR-221, miR-222. The multi-potent miRNA sponge inhibit cancer cell migration partially through the upregulation of Foxo3a,PTEN. Moreover they found that the antitumor function of the multi-potent miRNA sponge is much stronger than single miRNA targeting sponge and the four miRNAs used in this study had oncogenic functions in CRC. Future utility of the multi-potent miRNA sponge in the CRC treatment will be a promising and effective strategy. Being different from miRNA sponge,miRNA masking technology is developed by Choi et al. [141]. It consists of single-stranded 2’-O-methyl-modified antisense oligonucleotides that can fully bind to the 3’UTR of the target mRNA. One of the advantage of this technology is off-target effect can be significantly reduced which attracts the researchers’ attention in the CRC treatment. The screen of small molecule inhibitors of miRNA is being rapidly developed. Tripp et al. discovered small molecule inhibitors of miR-122 could be applied in the HCV therapy [142]. This novel approach combining other conventional CRC cancer therapeutics will play important roles in the future.

Another strategy to provide preclinical tools is miRNA restoration. Several miRNAs acting as tumor suppressors are generally downregulated in tumors. It has been demonstrated that the inhibition of tumor growth and angiogenesis is detected in xenografts of miR-27b mimics [143]. The utility of miRNAs mimics will provide a great clinical value for targeted therapies that identifies the cancer-related regulators. PH sensitive systemic administration of carbonate apatite nanoparticle-formulated miR-4689 reveals dramatically the inhibition of tumor growth in mouse xenografts with decreasing MAPK/ERK and PI3K/AKT signaling pathways [144]. In vitro colon cancer cells and in vivo mice bearing hepatic metastases models have been employed to test the tumor suppressor activity of miR-483-5p and miR-551a delivered by adeno-associated viruses (AAV) [97]. MiR-34a−/− mice have displayed an increased incidence and size of tumor with AOM/DSS challenge [95]. The expression of miR-34a is inhibited by specific antagomirs, a single strand RNA complementary to the targeted miRNA, which enhances the invasion of CRC cancer cells. However, ectopic expression of miR-34a can prevent IL-6-indiced EMT and invasion in DLD-1 cells. Likewise, miR-143 was found to be dramatically downregulated in the human CRC tissues as a tumor suppressor miRNA. Ng et al. increased miR-143 expression by transfection with miR-143 precursor in colon cancer cells [145], and found that restoration of miR-143 not only inhibited tumor cell growth but also affected malignant transformation phenotypes. Nakagawa et al. reported the increased expression level of miR-143 by α-mangostin induced human colon cancer DLD-1 cell death [146]. Taken together, restoring miRNA-based delivery systems as viable paths clinically is able to control cancer progression in cell tests and mouse models without any adverse outcomes.

A major obstacle to successful treatment for cancer is resistance to chemotherapy and radiation. Recently, miRNAs are being investigated as a predictor or a therapeutic target to improve the efficacy of 5-FU chemotherapy in CRC treatment. Various studies have shown that treatment with miR-22, miR-129, miR-365 and miR-143 increase sensitivity to 5-FU treatment in vitro and in vivo [73, 74, 147, 148]. However, high expression of miR-21 significantly decreases G2/M arrest and apoptosis after 5-FU treatment [137]. Silencing miR-21 inhibits cell proliferation and restores sensitivity of chemotherapy in HT-29 cells [149]. Moreover, miR-23a increases the chemoresistance to 5-FU in CRC cells though targeting ABCF1 [150]. A miRNA array screening revealed that miR-203 was significantly accumulated in oxanliplatin-resistant CRC cell lines [151]. Oxaliplatin is known to induce cell cycle arrest and cell apoptosis with a combination of therapeutic regimen for patients with metastatic CRC. In addition, the greater sensitivity to radiation is found in the treatment of miR-124 mimics to CRC cells and in the miR-124-overexpressed cells among in vivo mouse xenografts [152]. Therefore, understanding the miRNA-regulating mechanisms of resistance to chemotherapeutic agents would ultimately help us in improving therapeutic outcomes and identifying new targets and drugs.

## Conclusions

CRC is one of the most common malignancies in human. For patients with advanced CRC, the optimal treatment strategies currently depend on tumor staging and metastasis to reduce the risk of recurrence [153]. Resectable CRC is supported by combination with chemotherapy and non-resectable CRC, the systemic therapy options involve in palliative chemotherapy and monoclonal antibodies. However, more effective treatments with less cumulative toxicity and drug resistance are urgently needed.

The roles of miRNAs in tumor growth and the regulation of tumor progress summarized here suggest miRNAs could be a potential means for diagnosis and treatment of CRC as well as prognostic parameters for CRC. Future investigations will highlight the disease-specific or cell-specific expression patterns of miRNAs in CRC, which will be helpful to identify novel potential targets and improve our understanding of miRNA regulatory mechanisms. Moreover, extracellular miRNAs associated with cancer cells has recently emerged as new topic to explore and will expand the knowledge of tumor microenvironment modulation in CRC.
